# Proposal of a Methodology for Prediction of Indoor PM_2.5_ Concentration Using Sensor-Based Residential Environments Monitoring Data and Time-Divided Multiple Linear Regression Model

**DOI:** 10.3390/toxics11060526

**Published:** 2023-06-12

**Authors:** Shin-Young Park, Dan-Ki Yoon, Si-Hyun Park, Jung-In Jeon, Jung-Mi Lee, Won-Ho Yang, Yong-Sung Cho, Jaymin Kwon, Cheol-Min Lee

**Affiliations:** 1 Department of Chemical and Environmental Engineering, Seokyeong University, Seoul 02713, Republic of Korea; tlsdud060900@skuniv.ac.kr (S.-Y.P.); hhzz01@skuniv.ac.kr (J.-I.J.);; 2 Department of Nano and Biological Engineering, Seokyeong University, Seoul 02713, Republic of Korea; ydk0207@skuniv.ac.kr (D.-K.Y.); shp8880@skuniv.ac.kr (S.-H.P.); 3 Department of Health, Division Chemical Analysis Center, Korea Conformity Laboratories, Seoul 08503, Republic of Korea; 4 Department of Occupational Health, Daegu Catholic University, Gyeongsan 38430, Republic of Korea; whyang@cu.ac.kr; 5 Department of Nano, Chemical and Biological Engineering, Seokyeong University, Seoul 02713, Republic of Korea; yscho72@skuniv.ac.kr; 6 Department of Public Health, California State University, Fresno, CA 93740, USA

**Keywords:** indoor PM_2.5_, dwelling, outdoor variables, time, multiple linear regression, prediction model

## Abstract

This study aims to propose an indoor air quality prediction method that can be easily utilized and reflects temporal characteristics using indoor and outdoor input data measured near the indoor target point as input to calculate indoor PM_2.5_ concentration through a multiple linear regression model. The atmospheric conditions and air pollution detected in one-minute intervals using sensor-based monitoring equipment (Dust Mon, Sentry Co Ltd., Seoul, Korea) inside and outside houses from May 2019 to April 2021 were used to develop the prediction model. By dividing the multiple linear regression model into one-hour increments, we attempted to overcome the limitation of not representing the multiple linear regression model’s characteristics over time and limited input variables. The multiple linear regression (MLR) model classified by time unit showed an improvement in explanatory power by up to 9% compared to the existing model, and some hourly models had an explanatory power of 0.30. These results indicated that the model needs to be subdivided by time period to more accurately predict indoor PM_2.5_ concentrations.

## 1. Introduction

Koreans spend around 20.66 hours per day indoors, equivalent to spending more than 72 years in an indoor environment based on life expectancy as of 2021 [1,2]. As most modern people spend the majority of their time indoors, it is critical to identify and regulate the concentration of pollutants in the indoor environment. PM_2.5_ is particulate matter with an aerodynamic diameter of 2.5 μm or smaller and is detrimental to health due to its ease of adsorption and concentration of poisonous substances [3]. Previous studies have shown that long-term exposure to PM_2.5_ significantly increases the chances of cardiopulmonary problems and the mortality of lung cancers [4,5]. Recently, high concentrations of PM_2.5_ in the air have become common in Korea. Thus, it is necessary to know the spatial or temporal distributions of indoor PM_2.5_ concentrations to prevent adverse health impacts of PM_2.5_ exposure on occupants.

Indoor air quality is mostly monitored with measuring devices. However, assessment of measurement-based indoor air quality requires a significant amount of time and money. Among the various indoor spaces, difficulties in installing, maintaining, and repairing measurement devices are of particular note in residential environments [6]. To overcome the limitations of this measurement-based monitoring, many attempts have been made to estimate indoor particulate matter (PM_10_, PM_2.5_) concentrations. In particular, approaches through artificial intelligence, such as machine learning and deep learning, are being intensively researched [7,8]. Models that have been commonly used in these studies are multiple linear regression (MLR), decision tree model, support vector machine, random forests, and artificial neural networks (ANN) [9]. However, these prediction models only estimate parameters based on knowledge of other parameters (temperature, relative humidity, occupants’ activities, etc.) and are not designed to incorporate time as a variable or reflect temporal characteristics [9]. 

Meanwhile, since indoor air quality is affected by various factors, it is necessary to determine the relative contribution between variables affecting indoor PM_2.5_ concentration and determine input variables to accurately predict indoor air quality. Thus, previous studies have developed prediction models by choosing ventilation conditions, indoor pollutants, indoor airflow, and pressure that can be directly measured or validated indoors as input variables to increase predictive performance [10,11]. Using input variables obtained through sampling or surveys, such as ventilation rate, indoor pollutant concentration, pressure, and airflow, activity pattern information, may result in high performance [12]. However, these variables are difficult to obtain in real-time, making it even more challenging to predict indoor air quality accurately. 

To create an effective indoor concentration prediction model, it should be composed of easily obtainable variables. According to a previous study, it was found that indoor air quality was more affected by outdoor air quality than indoor sources in the case of natural ventilation [13]. When the windows were closed, outdoor sources accounted for 53 to 63% of indoor PM_2.5_ concentration. However, this increased to 92% when the windows were open [14]. In particular, the concentration of PM_2.5_ in outdoor air can enter indoors through cracks or gaps in building envelopes and windows, so outdoor PM_2.5_ is a key factor that can affect indoor PM_2.5_ [15].

In most previous studies, outdoor PM_2.5_ concentrations used fixed station’s data that were somewhat distant from the target point [16,17]. In the case of Korea, the national monitoring network is established at the city and count, so the outdoor PM_2.5_ concentration may differ from the indoor concentration in the air near the measured point [18]. By contrast, the I/O ratio, which is calculated as the ratio of the average indoor concentration to the average outdoor concentration, can estimate the approximate average indoor concentration through the outdoor concentration [19]. The data used to calculate the I/O ratio in most studies were performed simultaneously at the outdoor measurement point near the indoor sampling location [20]. Using the I/O ratio to estimate indoor PM_2.5_ concentrations can be a useful approach when it is difficult to obtain direct indoor PM_2.5_ measurements. However, it is important to note that the I/O ratio may not accurately reflect the actual indoor concentration as it assumes that the infiltration of outdoor PM_2.5_ is constant over time. An influencing variable of indoor PM_2.5_ concentration is only the outdoor PM_2.5_ concentration. Therefore, the I/O ratio method should be used with caution and supplemented with other methods to accurately predict the indoor PM_2.5_ concentration.

The main goal of this study was to overcome the limitations of using fixed station data as input values, which failed to reflect the temporal characteristics of existing indoor air quality prediction models. To achieve this, we used the I/O ratio method and utilized outdoor PM_2.5_ concentration, temperature, and humidity data measured near the indoor target point as input data to investigate the relationship between indoor and nearby outdoor PM_2.5_ concentrations. Subsequently, the indoor PM_2.5_ concentration was calculated using a multiple linear regression (MLR) model, employing easily available variables such as meteorological data, and the influence of outdoor variables was confirmed through weights and error terms. Finally, our aim was to provide an easily utilizable indoor PM_2.5_ concentration prediction method that can accurately reflect temporal characteristics.

## 2. Materials and Methods

### 2.1. Measurement Method

In this study, we measured the indoor and outdoor PM_2.5_ concentrations, temperature, and relative humidity for our indoor PM_2.5_ concentration prediction model. Other meteorological variables were obtained from the automatic weather system (AWS) data provided by the Korea Meteorological Administration. 

**Figure 1:**
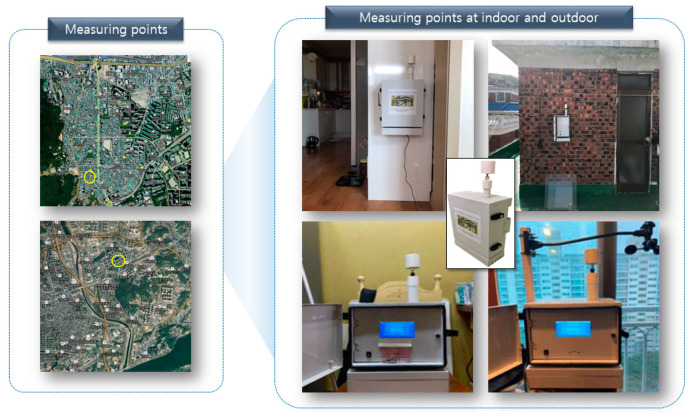
Location (yellow circle) of the monitoring device.

First, measurable variables (PM_2.5_ concentration, temperature, and relative humidity) were collected from the inside and outside of a house in Bu-Cheon and Nam-Yang-Ju. The measuring point was located within a residential complex, with the Si-Heung interchange of the Metropolitan First Circular Expressway approximately 1.8 km to the south and Nam-Yang-Ju interchange of Metropolitan First Circular Expressway and North Arterial Road approximately 1 km to the west and north (Figure 1).

An outdoor air quality measuring instrument (Dust Mon, Sentry Co. Ltd., Seoul, Republic of Korea) using light scattering was used. This instrument had an error within 80–120% of the total average value of PM_2.5_ measured using standard equipment based on variability evaluation among monitoring equipment at the Korea Testing & Research Institute. The specifications of the measuring instrument are shown in Table 1, and the flow rate was fixed at 0.5 L/min. Measurement data were collected in real-time through LTE Cat M1 and stored on an SD card within the instrument. The instrument was attached to the roof of the third floor of the target house and to the wall of the living room in Bu-Cheon and to the balcony, and to the room in Nam-Yang-Ju. Indoor and outdoor real-time PM_2.5_ concentrations were measured simultaneously at one-minute intervals for one-year (1 May 2019 to 30 April 2020 and 27 June 2020 to 22 April 2021).

The meteorological data utilized in the study were obtained from AWS located at the target points in Bu-Cheon (37°50′05.49″ N, 126°76′36.40″ E) and Nam-Yang-Ju (37°63′42.48″ N, 127°15′11.84″ E).

### 2.2. Data Analysis 

#### 2.2.1. Statistics Analysis

Indoor and outdoor variables, including PM_2.5_, temperature, and relative humidity, were directly measured and collected at ten-minute intervals. The meteorological data (wind direction, wind speed, precipitation) were obtained through an AWS, and atmospheric data and time data (year-month-day 00:00) were extracted by averaging the minute data over ten-minute intervals. Rows containing missing or negative values were removed before performing descriptive statistical analysis and correlation analysis using the statistical program R.

Spearman’s rank correlation analysis, a non-parametric analysis method, was used for the correlation analysis considering the non-normal distribution of PM concentrations, and the significance level was set at 0.05. 

#### 2.2.2. Selection of Input Variables

The selection of input variables is a crucial consideration in modeling methods because they determine the model structure and can impact the coefficients and overall performance [21,22,23]. In this study, we aimed to use easily accessible meteorological variables as input variables. However, these variables exhibit nonlinear characteristics when predicting PM concentration [24]. This is due to numerous artificial conditions, such as household heating, transportation, and the activities of occupants, that can affect the immediate PM concentration [25]. Although using corresponding variables to increase the predictive power of the model may seem effective, it is not feasible for the purpose of this study, which is to propose an effective model. Furthermore, since sources that affect particulate matter concentration differ across regions, applying a variable that can impact immediate PM concentration is limited to a specific location.

In this study, outdoor PM concentration, temperature, relative humidity, and other meteorological variables were selected as the main input variables. Additionally, to overcome the limitation of the existing prediction model’s training data being somewhat distant from the indoor target point, a measurement method was applied to calculate the I/O ratio. Data obtained from measuring devices installed outdoors near the target point were used to determine the outdoor PM_2.5_ concentration, temperature, and relative humidity.

The MLR model used in this study is based on the I/O ratio, with the outdoor PM_2.5_ concentration selected as the initial variable. Other variables, including indoor and outdoor temperature and relative humidity measured by a sampling device, AWS data (wind speed, wind direction, and precipitation), as well as the difference between indoor and outdoor temperature and relative humidity, were also selected as input variables. Currently, the temperature and relative humidity difference between indoor and outdoor environments are recognized as contributing factors to natural ventilation, and hence, it was considered as a potential input variable [26]. The final input variables were chosen based on the results of correlation analysis and previous research. 

To check for multi-collinearity among the independent variables, the variance inflation factor (VIF) was calculated using Equation (1). A VIF value of 1 indicates independence among variables, while a value > 5 indicates a high correlation among variables. If the VIF value is >10, one of the variables violating independence must be removed [27].

(1) $$VIF_{i}=\frac{1}{1-R_{i}^{2}}$$

In Equation (1),$VIF_{i}$ is the variance expansion factor for the ith independent variable, and $R_{i}^{2}$ is the R^2^ value of regression analysis after removing the ith independent variable.

### 2.3. Data Preprocessing before Training

**Figure 2:**
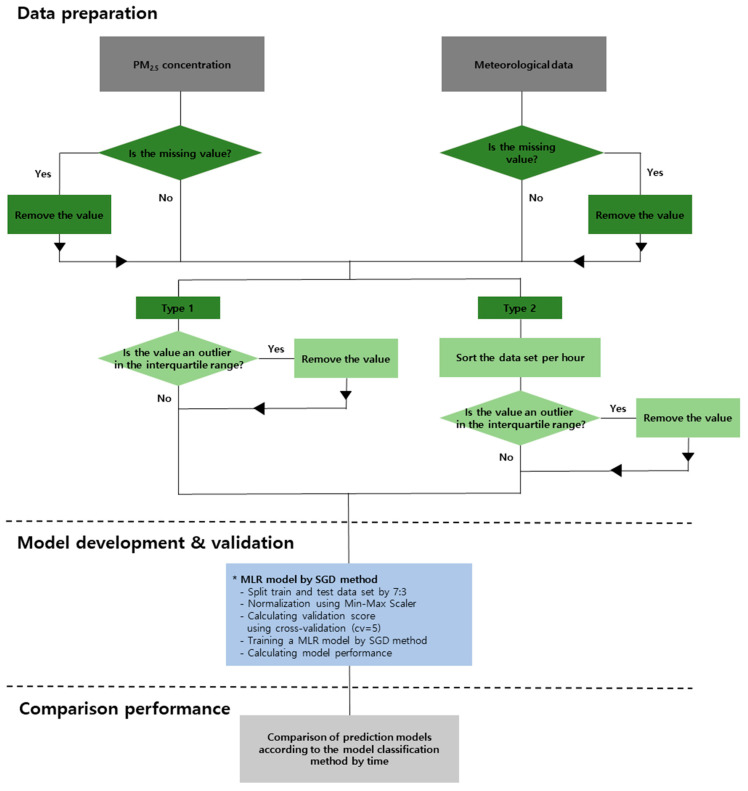
The flowchart of data pre-processing, model development and validation, comparison performance.

Data pre-processing and learning methods are shown in Figure 2. The dataset was first processed to remove missing values, after which it was divided into hourly units based on date and time, resulting in 24 datasets from 0:00 to 23:00. To ensure that the model predicts a universal level of concentration and improve its performance, outliers of indoor PM_2.5_ concentration were removed from the 24 datasets using the interquartile range method [28]. The equations for calculating the interquartile range (Equation (2)) and for detecting outliers (Equation (3)) are as follows:

(2) $$IQR=Q_{3}-Q_{1}$$

(3) $$Q_{1}-1.5\times IQR\leq x\leq Q_{3}+1.5\times IQR$$

In Equations (2) and (3), IQR denotes the interquartile range, $Q_{3}$ denotes the third quartile, and $Q_{1}$ denotes the first quartile. 

### 2.4. Multiple Linear Regression Model

Multiple linear regression (MLR) is a technique used for modeling the linear relationship between two or more variables. The model is fitted such that the sum of squares of differences between observed and predicted values is minimized [29]. The following represents an MLR model:

(4) $$y=a_{1}x_{1}+a_{2}x_{2}+a_{3}x_{3}+\cdots {+a}_{n}x_{n}+\varepsilon$$

In Equation (4), y is the dependent variable (PM_2.5_), $x_{1}$, $x_{2}$, $x_{n}$ are independent variables, ε is the intercept.

MLR was used to create a prediction model for indoor PM_2.5_ concentration. Prior to the application of the MLR procedure, all data were normalized according to Equation (4) [30].

(5) $$Z_{i}=\frac{x_{i}-min(x)}{\max\left(x\right)-min(x)}$$

In Equation (5), $Z_{i}$ denotes ith normalized value, $x_{i}$ denotes ith observed value for the variable x, min(x) denotes minimum value in the dataset, max(x) denotes maximum value in the dataset. MLR model was formulated using Scikit-learn (version 1.0.2) [31].

In this study, we aim to develop a real-time indoor PM_2.5_ concentration prediction model using gradient descent, which is a method used for estimating model weights in deep learning models such as neural networks.

The gradient descent methods include full gradient descent (i.e., batch gradient descent), stochastic gradient descent (SGD), and mini-batch gradient descent [32]. Full gradient descent uses the entire dataset to update the parameters once, but it can take a long time to calculate the coefficients if the dataset is large. In the case of the SGD method, an appropriate gradient can be obtained for one data point that has been randomly sampled from all the data to update the weight quickly. With mini-batch gradient descent, the gradient is calculated using a randomly selected batch size [33]. This method is often used because it is known to solve the problem of gradient vanishing and exploding, which can prevent finding better weight. However, very recently, it has been discovered that mini-batching is not necessary to resolve the non-vanishing variance issue inherent in the original SGD methods [31]. 

The regression coefficient of the prediction model was calculated using the SGD method and scikit-learn’s “SGD-Regressor” library for training. Default values were used for hyper-parameters that could not be adjusted, except for the regularization intensity (alpha) and initial learning rate (eta0) parameters [31].

Regularization is a method used to prevent the overfitting of MLR. Ridge regression (L2), a type of weight regularization method, was applied to utilize all the selected variables. Ridge regression includes a penalty term, as shown in Equation (6), which helps to improve the overfitting problem of the model by adjusting the alpha value of the penalty term to reduce the overall weight. The larger the alpha value, the stronger the regularization intensity; when alpha is zero, regularization is not applied. To apply an appropriate regularization intensity, we adjusted the alpha value to a multiple of 10 within the range of 0.0001 to 10.

(6) argminw,b1n∑i=1n(yi−yi^)2

(7) Error=argminw,b1n∑i=1n(yi−yi^)2+awi2

where $y_{i}$ is the measured value, $\hat{y_{i}}$ is the predicted value, n is the number of data, $w_{i}^{2}$ is the weight and a is alpha. 

Meanwhile, the initial learning rate (eta0) was set to 0.001 instead of the default value of 0.01. This change was made because when eta0 was set to the default value, it converged to local optimization instead of global optimization.

The dataset was split into 70% training data and 30% test data for use in the SGD model. Random classification was applied using the ‘train_test_split’ and ‘random_state’ libraries in the scikit-learn package. The SGD models were trained using the training dataset, and their performance was assessed using the testing data that were not used during training.

### 2.5. Performance Indicators

The accuracy of the MLR methods was evaluated using the coefficient of determination ($R^{2}$), root-mean-square error (RMSE), and mean absolute error (MAE). The R^2^ value is commonly used to explain how much of the variability in the predicted data can be explained by the relationship between the predicted and observed values. The RMSE and MAE are used to measure the difference between the measured and predicted values. The equations for these performance indicators are given in Equations (8)–(10), respectively [34]:

(8) $$R^{2}=1-\frac{RSS}{TSS}=1-\frac{\sum_{i=1}^{n}(y_{i}-\hat{y_{i}}{)}^{2}}{\sum_{i=1}^{n}(y_{i}-\bar{y}{)}^{2}}$$

(9) $$RMSE=\sqrt{\frac{\sum_{i=1}^{n}(y_{i}-\hat{y_{i}}{)}^{2}}{n}}$$

(10) $$MAE=\frac{\sum \left|y_{i}-\hat{y_{i}}\right|}{n}$$

where $y_{i}$, $\hat{y_{i}}$, and $\bar{y}$ are the measured and predicted values of each output variable, and n is the number of samples.

## 3. Results

### 3.1. Distribution Characteristics of Indoor and Outdoor Measurement Data

As a result of identifying the distributions of measurement data, indoor PM_2.5_ concentration was 10.31 ± 13.70 μg/m^3^, the outdoor PM_2.5_ concentration was 26.28 ± 20.69 μg/m^3^, and the I/O ratio was 0.39 and median ratio was 0.29 (Table 2). To investigate the distribution of each variable, skewness, and kurtosis were calculated. As a result, indoor PM_2.5_ concentrations were found to have positively skewed and highly peaked distributions, with skewness values of 4.44 and kurtosis values of 50.27. Similarly, outdoor PM_2.5_ concentrations were found to have similar distributions. By contrast, temperature and relative humidity were found to have approximately normal distributions.

**Figure 3:**
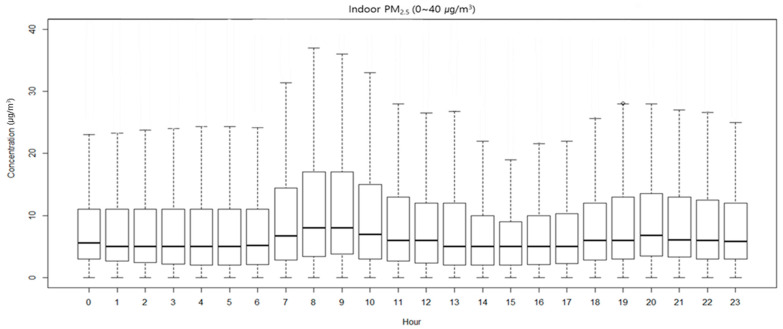
Distribution of indoor PM_2.5_ by the hour.

This study aimed to develop an indoor PM_2.5_ concentration prediction model that reflects temporal characteristics. Thus, the distribution characteristics of indoor PM_2.5_ concentrations by time were determined (Figure 3). Indoor PM_2.5_ concentrations were higher between 7–10 h and 19–21 h than at other times. Furthermore, it was verified that an extreme concentration compared to the average indoor PM_2.5_ concentration appeared in the evening (17–20 h).

### 3.2. Selection of Input Variables

**Figure 4:**
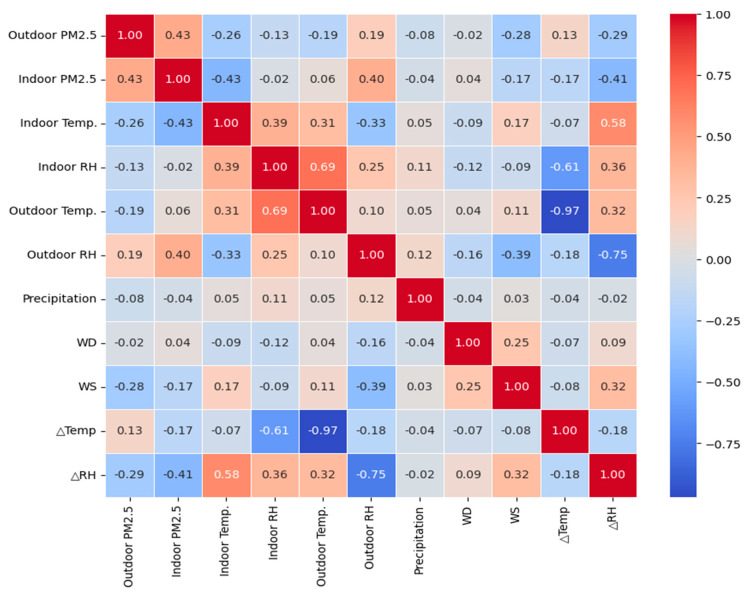
Heatmap of variables.

To select input variables that can predict indoor PM_2.5_ well, the correlation coefficient was checked (Figure 4). Indoor PM_2.5_ concentration was found to have the highest correlation with PM_2.5_ (r = 0.43) among outdoor parameters, followed by relative humidity (r = 0.40) and wind speed (r = −0.17) (*p* < 0.05). In the case of indoor parameters, temperature (r = −0.43) (*p* < 0.05) had a high correlation. The indoor/outdoor temperature difference had a weak negative correlation (r = −0.17), and the relative humidity difference had a negative correlation (r = −0.41) (*p* < 0.05).

Initially, input variables with a correlation coefficient of 0.1 or higher were selected from the available variables [35]. Then, VIF, a multicollinearity indicator, was used to select the final set of input variables. The VIF values were 9.38 for outdoor PM_2.5_, 2.33 for indoor temperature, 1.28 for wind speed, 3.75 for outdoor relative humidity, 1.32 for temperature difference (ΔTemp), and 5.33 for relative humidity difference (ΔRH), due to the concern of multicollinearity. Finally, the selected input variables were outdoor PM_2.5_, indoor temperature, wind speed, outdoor relative humidity, temperature difference (ΔTemp), and relative humidity difference (ΔRH).

### 3.3. Model Training Result

#### 3.3.1. MLR Model

The result of the training MLR model by the previous method is summarized in Table 3. As a result of checking the model’s performance, the explanatory power of the model that did not reflect time series characteristics was found to be 25%, and the RMSE and MAE were 4.87 and 3.66, respectively. Furthermore, as a result of checking the weight of the model, it was confirmed that the outdoor PM_2.5_ concentration had the greatest effect on the indoor PM_2.5_ concentration (16.44), and the indoor temperature was found to have the next negative effect (−9.44). 

The calculated regression coefficients and error terms are as follows:

(11) $${PM}_{2.5_{in}}=a\cdot {PM}_{2.5_{out}}+b\cdot {Temp.}_{in}+c\cdot {RH}_{out}+d\cdot {WS}_{out}+e\;\cdot △Temp.+\;f\;\cdot △RH+\varepsilon$$

where a, b, c, d, e, and f denote regression coefficients and the measured and predicted values of each output variable, and ε denote error terms.

#### 3.3.2. MLR Model Divided by Hour

At this time, unlike the previous model, the prediction model separated the dataset per hour, removed outliers using the interquartile range, and calculated the prediction model through the pre-processed dataset. The prediction model for indoor PM_2.5_ concentration separated per hour was learned (Table 4).

The explanatory power of the model divided into time periods was determined to be about 20~34%, and the RMSE and MAE were confirmed to be 4~7 and 3~5, respectively. The explanatory power of the time-specific model was found to be improved by up to 9%, and the error between the measured value and the predicted value was improved.

The explanatory power of each model divided per hour was the highest at 0.34 for H4 (4:00~4:59) models. The RMSE and MAE of the H15 (15:00~15:59) model were 3.34157 and 2.55109, respectively, showing the smallest error between the measured value and the predicted value.

At this time, unlike the previous model, the prediction model separated the dataset per hour, removed outliers using the interquartile range, and calculated the prediction model through the pre-processed dataset. The prediction model for indoor PM_2.5_ concentration separated per hour was learned (Table 5).

As a result of checking the regression coefficient of the MLR model, it was established that the variable that had the greatest effect on the indoor PM_2.5_ concentration was the indoor temperature at the H9 model, and it was confirmed that it had the greatest negative (−) effect at −17.11. The outdoor PM_2.5_ was found to have the next largest positive (+) effect, with 15.70 at H8.

The calculated regression coefficients and error terms are as follows:

(12) $${PM}_{2.5_{in}}=a_{t}\cdot {PM}_{2.5_{out}}\left(t\right)+b_{t}\cdot {Temp.}_{in}\left(t\right)+c_{t}\cdot {RH}_{out}\left(t\right)\;+d_{t}\cdot {WS}_{out}\left(t\right)+e_{t}\;\cdot △Temp.(t)+f_{t}\;\cdot △RH(t)+\varepsilon_{t}$$

where $a_{t}$, $b_{t}$, $c_{t}$, $d_{t}$, e, and $f_{t}$ denote regression coefficients, and the measured and predicted values of each output variable, and $\varepsilon_{t}$ denote error terms.

## 4. Discussion

### 4.1. Indoor PM_2.5_ Concentration and Outdoor Variables

The study aimed to propose a method for predicting indoor PM_2.5_ concentration by time by adding meteorological variables based on the I/O ratio method using PM_2.5_ concentration data measured in outdoor air adjacent to indoors. To confirm the correlation between indoor and outdoor PM_2.5_ concentrations, PM_2.5_ was measured in outdoor air adjacent to indoors. The outdoor PM_2.5_ concentration at the study site was found to be 26.28 ± 20.69 μg/m^3^, which is approximately twice the domestic annual average standard of 15 μg/m^3^. This concentration is also 1.5 times higher than the annual average PM_2.5_ concentration (20 μg/m^3^) of Seoul in 2021, according to the Air Quality Annual Report of the Ministry of Environment [36]. The indoor PM_2.5_ concentration was determined to be 14.86 ± 15.23 μg/m^3^, which is lower than the standard (35 μg/m^3^) for facilities used by sensitive classes in the indoor air quality management standard [37].

As a result of checking the indoor PM_2.5_ concentration characteristics by time zone, the highest concentration was 14.51 µg/m^3^ at 9:00, and the PM_2.5_ concentration between 7 and 10 o’clock was higher than at other times. In the afternoon, it gradually increased after 18:00, appeared high at 11.62 µg/m^3^ at 20:00, and then gradually decreased. These results were similar to those of previous studies [38,39,40,41,42,43]. The indoor PM_2.5_ concentration in the morning was higher than at other times, which could be due to occupants preparing for the day, cooking breakfast, or opening windows for ventilation. In the evening, the PM_2.5_ concentration gradually increased, which could be due to occupants returning home and cooking dinner. The outdoor PM_2.5_ concentrations on weekdays near apartments gradually increased from 6:00 a.m. and peaked at 9:00 a.m. [39]. It is assumed that this was due to the fact that car traffic and population movement during commuting hours affect the outdoor PM_2.5_ concentration in locations where residential complexes are concentrated, such as the study site.

After conducting a correlation analysis between indoor PM_2.5_ and outdoor PM_2.5_, it was found that the concentration of indoor PM_2.5_ had a correlation coefficient greater than 0.43 with the outdoor PM_2.5_ concentration. However, these results showed a lower correlation with outdoor PM_2.5_ compared to previous studies [40,41,42,43,44]. Meanwhile, in a dry urban environment where atmospheric dust events frequently occur, the correlation between indoor and outdoor PM_2.5_ (r = 0.82) was confirmed to be very high even when the windows were closed [41]. These findings suggest that the indoor PM_2.5_ concentration can also increase when the wind speed is strong or when the PM_2.5_ concentration in the outdoor air is high. In addition, it was also validated that the outdoor PM_2.5_ concentration had a quantitative effect on the indoor PM_2.5_ concentration even with the windows closed in offices in downtown areas where dust storms did not frequently occur [39]. These results suggest that the outdoor PM_2.5_ concentration can be used as an indicator to predict the indoor PM_2.5_ concentration and that the correlation between indoor and outdoor PM_2.5_ concentrations can be improved by considering the following meteorological variables with wind speed and direction.

Moreover, based on the analysis, indoor PM_10_ had the highest correlation coefficient of 0.95, followed by outdoor PM_10_, outdoor PM_2.5_, indoor temperature, ΔRH, outdoor RH, wind speed, and ΔT, in that order (*p* < 0.05). Excluding indoor PM_10_, significant correlations were found between outdoor PM_10_ and PM_2.5_ concentrations with indoor PM_2.5_ and indoor temperature also showed a significantly negative correlation with indoor PM_2.5_ concentrations (r = −0.43). Additionally, a significantly high positive correlation of 0.40 or more was observed between outdoor RH and ΔRH.

In other words, meteorological conditions can significantly impact indoor PM concentration levels, as they can affect the particle size depending on the indoor air exchange rate, relative humidity, and the origin of the air mass [45,46]. Therefore, it can be inferred that outdoor meteorological variables may be used to predict indoor PM_2.5_ concentrations.

### 4.2. Previous Studies about Indoor PM_2.5_ Concentration Prediction Model

In this study, the MLR model was used to predict indoor PM_2.5_ concentration, and the model was subdivided by time period. The results of previous studies that used the MLR model to predict indoor PM_2.5_ concentration are presented in Table 6. Three out of five studies used survey results as indoor variables, including questions on the number of pets, rooms, air purifiers, use of air fresheners, occupant activity patterns, and building characteristics [36,46,47]. However, the model that was mainly composed of survey items had an explanatory power of 0.35, which was lower than other studies. Other input variables used in these studies included indoor PM_10_, PM_2.5_ concentration, temperature, relative humidity, and ventilation rate [36].

All studies have verified that outdoor PM_2.5_ concentration is a crucial input variable in predicting indoor PM_2.5_ concentration, using outdoor-related variables such as PM_10_, PM_2.5_ concentration, temperature, relative humidity, wind speed, NO_2_, CO_2_, etc. [46,47,48,49]. Therefore, it can be inferred that outdoor PM_2.5_ concentration plays a significant role in determining indoor PM_2.5_ concentration.

In this study, the prediction model was subdivided by time period. Although no study subdivided the model by time period, as in our study, one study classified it by season, showing that the explanatory power of each model differed by season, and the annual model performed the best. However, this result was influenced by the dataset size, which is an important parameter for model evaluation. Furthermore, the regression coefficient of outdoor PM_2.5_ in the MLR model varied by season, with autumn (0.58), winter (0.69), spring (0.69), and annual (0.88) seasons showing different effects on indoor PM_2.5_ [48].

Most studies used data measured for a short period of time, ranging from 48 h to four months, for model training rather than data collected over a year. Additionally, one study used outdoor data from a national measurement network that was distant from the indoor measurement point. Some studies measured both indoor and outdoor air quality at nearby locations simultaneously, as in our study, but most used data were measured for a short period of time.

The performance of the prediction model was evaluated, and the model that used data measured in the experimental building had the lowest RMSE (0.09) and highest R^2^ (0.99) values, followed by the model predicting PM_2.5_ concentration in school indoor spaces [48,49]. However, the model developed for residential interiors had an explanatory power of less than 50% based on the validation score [36,46,47]. This is likely due to the significant variation in indoor pollutant concentrations based on individual occupant characteristics, as well as the presence of various sources in living spaces, unlike laboratory buildings or schools. Nevertheless, even when using input variables such as direct measurements or survey results for indoor pollutant concentrations, ventilation rates, or other factors, similar explanatory power to the time zone-based model proposed in this study was observed [36,46,47]. It is expected that the model could, to some extent, reflect the activity patterns of occupants through further subdivision.

**Table 6:** Summary of indoor PM_2.5_ prediction studies using MLR model.

Indoor Type	Variable		Time Division (O/X) ^(1)^	Data	RMSE	R^2^	Ref.
	Indoor	Outdoor					
Dwelling	Survey result	PM_2.5_	X	(1) Country: America (2) Sampling period: 48 h samping (3) Sampling the indoor and outdoor data simultaneously, nearby	-	0.35	[36]
Apartment	Survey results, building characteristics	PM_2.5_ concentration, temperature, wind speed	X	(1) Country: Mongolia (2) Sampling period: 7 days, during 24 h (3) Indoor data: The direct measurement of indoor air (4) Outdoor data: The national monitoring network	0.48, 0.50 (val) ^(2)^	0.52, 0.49 (val)	[46]
Dwelling	PM_10_2.5_, survey result, VOCs, building characteristics	PM_10_2.5_, RH, PM_2.5_	X	(1) Country: Japan (2) Sampling period: 7 days, during 24 h (3) Sampling the indoor and outdoor data simultaneously, nearby	15.70 (val)	0.42 (val)	[47]
School	Relative humidity, temperature, Ventilation	PM_2.5_, CO_2_, wind speed, PM_10_	*O*	(1) Country: Israel (2) Sampling period: 7 days, 7:00–12:00 in winter and spring, 12:00–17:00 in fall (3) Indoor and outdoor measurements alternately at 15 min intervals	0.17 (Fall), 0.13 (Winter), 0.14 (Spring), 0.08 (Annual)	0.58 (Fall), 0.69 (Winter), 0.69 (Spring), 0.88 (Annual)	[50]
Laboratory building	Temperature, Relative humidity, PM_10_, NO_2_	Temperature, Relative humidity, PM_10_, NO_2_, PM_2.5_	X	(1) Country: America (2) Sampling period: May-September 2020 during 24 h (3) Sampling the indoor and outdoor data simultaneously, nearby (4) Reflection of time delay effect (TSR model)	0.09	0.99	[51]

^(1)^ Time division (O/X): whether the model was divided by time (season, month, hour, etc.). ^(2)^ val: score of validation data.

### 4.3. MLR Model

In the MLR model, the higher the slope, the greater the influence on the dependent variable, and the larger the intercept, the larger the dependent variable on average [41]. In this study, outdoor PM_2.5_ concentration and environmental parameters were used as input variables for the indoor PM_2.5_ concentration prediction model, and normalized data were applied to the MLR model to regress the importance of each variable on indoor PM_2.5_ concentration.

As a result, the study found that the outdoor PM_2.5_ concentration had the greatest positive effect on the indoor PM_2.5_ concentration (16.44), followed by the indoor temperature (−9.44) having a negative effect. This can be interpreted as the indoor PM_2.5_ concentration increases as the outdoor PM_2.5_ concentration is high and the indoor temperature is low. It is judged to have influenced according to a previous study using variables similar to this study, outdoor PM_2.5_, wind speed, temperature, and relative humidity had an effect on the indoor PM_2.5_ concentration in the order, and it was verified that wind speed had a significant effect unlike in this study [41]. Unlike this study, which considered wind speed in all directions, it is judged that the difference in importance was caused by the use of wind speed in consideration of the wind direction affecting the research target point in the previous study. Additionally, the study found that PM_2.5_ in the air easily penetrates indoors through window cracks during winter [43].

**Figure 5:**
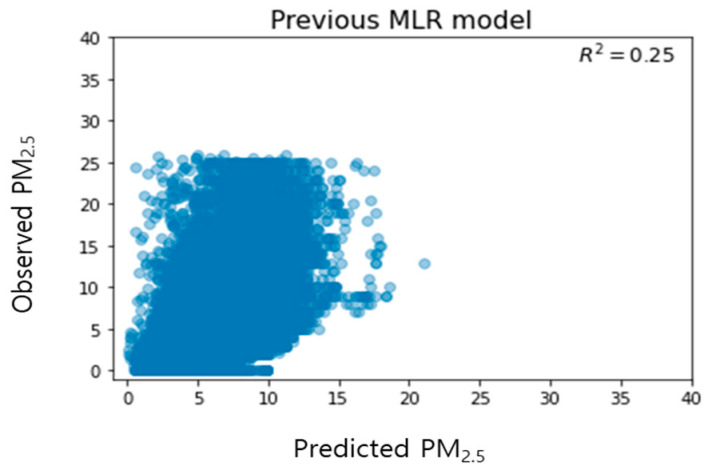
Relationship between predicted and observed indoor PM_2.5_ concentration according to the previous multiple linear regression models.

Figure 5 shows the distribution of measured and predicted values from the previous model. Considering that the predicted value and the measured value are tilted along the y-axis, it was confirmed that the predicted value was generally underestimated compared to the measured value.

On the other hand, it is known that the concentration of PM_2.5_ in indoor air is greatly influenced by the activity patterns of occupants and indoor sources [45,50,51,52]. However, in order to reflect factors such as indoor sources, survey results must be used, and survey results are difficult to obtain compared to outdoor variables. Meanwhile, indoor sources are heavily influenced by occupants’ activities, and according to previous research findings, except for special events, occupants’ daily activity patterns tend to exhibit similarity by time period [38,39,40,41,42,43]. In this study, in order to reflect the distribution characteristics of indoor PM_2.5_ concentration by time and activity patterns of indoor occupants, the model was subdivided by the hour, and the importance of variables in the model by time zone and the influence on indoor PM_2.5_ concentration by time zone were analyzed. In order to check the weight of the variable, the slope and intercept were checked.

As a result, indoor temperature had the greatest effect (–17.11) on indoor PM_2.5_ concentration, followed by outdoor PM_2.5_ concentration (15.70). This differed from the previous model, which did not differentiate between time zones, and highlighted the importance of segmenting the model. In the case of indoor temperature, it was confirmed that a significant negative effect was given between 8–10H models, and the outdoor PM_2.5_ concentration also showed a large weight between 7–10H models. Furthermore, as a result of checking the intercept, the 8–11H model appeared higher than other models, and it was found that the indoor PM_2.5_ concentration appeared high during that time [45]. This is a result that can verify that there is an indoor source in the corresponding time. In addition, the intercept of the 18H model was 8.55, which was higher than that of the before and after models. In the case of the residential environment, it was a result that could be estimated that the occupant’s cooking activity would be the main indoor source. Prior research has shown that cooking can double indoor PM_2.5_ exposure [53]. In addition, it was confirmed that the indoor PM_2.5_ concentration lasted for about 30–60 min during cooking [43]. However, in this study, considering that the indoor concentration is high for about 3–4 h in the morning, it is judged that there is an additional source, such as cleaning activity or the concentration of PM_2.5_ in the outdoor air is high during traffic congestion.

**Figure 6:**
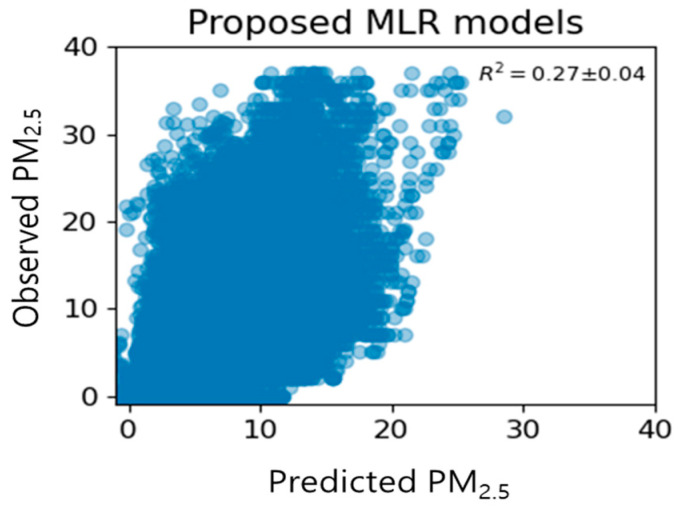
Relationship between predicted and observed indoor PM_2.5_ concentration according to the proposed multiple linear regression models (H0–H23).

Figure 6 shows the distribution of the measured and predicted values of the proposed time-specific model. After classification by time period and removing outliers, the model predicted a wider range of concentrations and showed slightly improved prediction performance compared to the existing model with an average of 27% ± 4%. The R^2^ values of Figure 5 and Figure 6 may appear to have little difference, but it is important to note that R^2^ can be greatly influenced by sample size. Therefore, the observed results are considered noteworthy. However, the predicted value was generally underestimated compared to the measured value, as seen by the tilt of predicted value and the measured values along the y-axis, similar to the existing model by time period.

**Figure 7:**
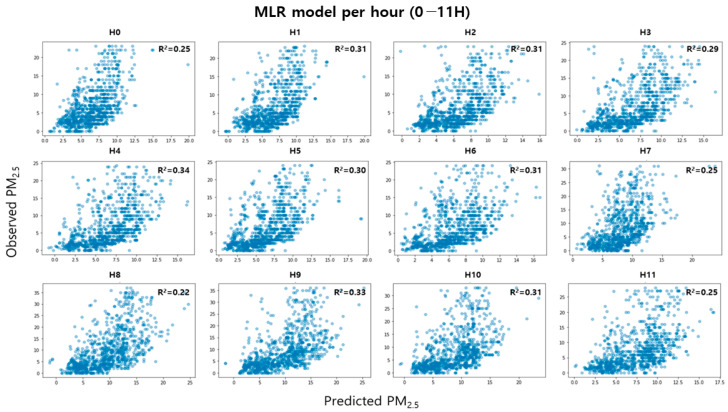
Relationship between predicted and observed indoor PM_2.5_ concentration per hour in a.m.

**Figure 8:**
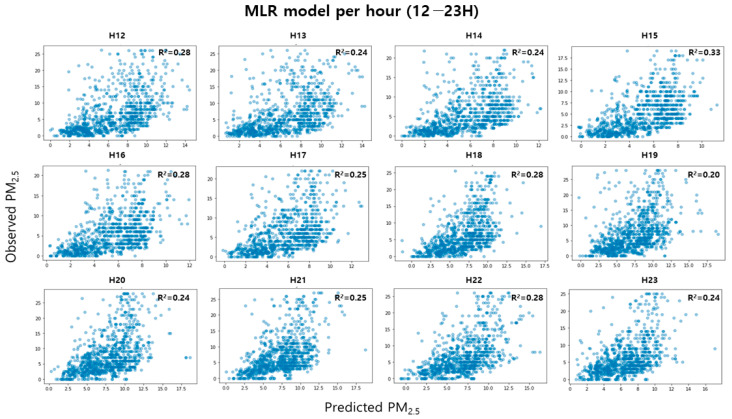
Relationship between predicted and observed indoor PM_2.5_ concentration per hour in p.m.

As a result of examining the performance of the MLR model classified by time unit, the explanatory power was improved by up to 9% compared to the existing MLR model. Figure 7 and Figure 8 are the test results for indoor PM_2.5_ concentration per hour; the explanatory power of the H1, H2, H4, H5, H6, H9, H10, and H15 models was 0.30 or higher, compared to the existing MLR model. These results show that since the distribution characteristics of indoor PM_2.5_ concentrations are different for each time period, a model with high accuracy can be developed only when the model is subdivided and trained in consideration of time. In addition, since it was applied to residential space with various indoor sources and different activity patterns of occupants, the method proposed in this study would have been applied to indoor spaces such as offices where the types of indoor sources were relatively few and the activity patterns of occupants were constant. It is considered that the predictive performance is better.

However, the explanatory power of the time zone-based classification model proposed in this study was relatively low compared to other studies, possibly due to the limited data used for model training which were collected from only two households. It is believed that a more accurate and generalized model can be developed by incorporating a variable that has a significant impact on indoor PM_2.5_ concentration. Since this study divided the model by time zones based on indoor PM_2.5_ concentration, it is expected that the limitations of direct measurement and investigation can be partially overcome by reflecting the distribution characteristics of indoor PM_2.5_ concentration across different time zones.

### 4.4. Influence of Seasonal Characteristics on Prediction Results of PM_2.5_ Cocentration

**Figure 9:**
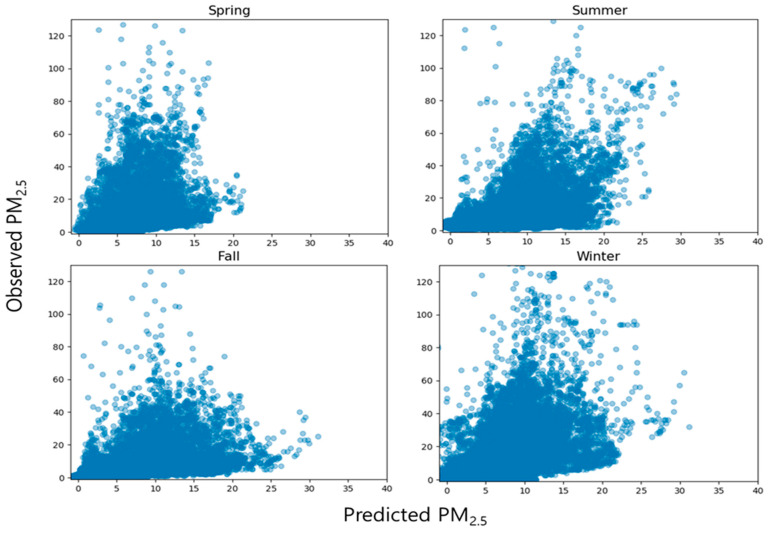
Relationship between predicted and observed indoor PM_2.5_ concentration according to the seasonal characteristic (H0–H23).

Our study trained an MLR model using PM_2.5_ concentration data collected simultaneously indoors and in the nearby outdoor areas, along with hourly meteorological data, over a period of one year. We categorized the dataset into four seasons: spring (March to May), summer (June to August), autumn (September to November), and winter (December to February). We aimed to examine the performance differences of the prediction model according to the seasons and determine the impact of seasons on indoor PM_2.5_ concentrations. We applied the categorized datasets to the prediction model and compared the results with the previously calculated test-RMSE of the model (Table 7). The distribution of predicted values and actual measurements was examined (Figure 9).

Among the four seasons, the RMSE values were found to be highest in the order of spring, winter, summer, and autumn. When evaluating the model performance based on different time periods, significant errors were observed in the predicted values compared to the actual values for H15 in spring and winter, H5 in summer, and H19 in autumn. Upon analyzing the input data of the models during the time periods with high errors, the maximum concentrations were found to be 171.85 μg/m^3^, 160.00 μg/m^3^ in both spring and winter, 126.00 μg/m^3^ in summer, and 344.40 μg/m^3^ in autumn, respectively. The substantial differences between these values and the maximum RMSE values observed during the corresponding seasons (spring—60.00 μg/m^3^, winter—72.00 μg/m^3^, summer—57.00 μg/m^3^, autumn—43.98 μg/m^3^) indicate the possible presence of indoor pollution sources during those specific time periods. These findings are expected to serve as important reference data for future studies on indoor PM_2.5_ concentration prediction models.

## 5. Conclusions

Most people spend much of their time indoors, and it is important to regulate indoor air quality to prevent health problems related to exposure to PM_2.5_. While measuring devices are commonly used to monitor indoor air quality, it can be difficult and expensive to assess measurement-based indoor air quality. This study aims to provide a more easily utilizable indoor PM_2.5_ concentration prediction method that can accurately reflect temporal characteristics by utilizing outdoor PM_2.5_ concentration, temperature, and humidity data measured near the indoor target point as input data to calculate indoor PM_2.5_ concentration through a multiple linear regression model.

To address the limitations of the MLR model and capture the distribution characteristics by time period, the dataset was divided into hourly units. Additionally, outliers were removed from the dataset during model training by utilizing the interquartile range to produce a more accurate and universally applicable concentration value.

As a result of the training, a significant difference in model performance was observed depending on whether or not the time zones were taken into account. By incorporating temporal characteristics into the training process, the MLR model showed an up to 9% improvement in explanatory power compared to the existing model. Some temporal models demonstrated an explanatory power of 30% or higher.

On the other hand, since the model was trained using data collected from two specific dwellings, its accuracy may be lower when applied to different indoor spaces. Furthermore, the explanatory power of the predictive model was relatively low due to the limited availability of input variables that could be easily obtained. In addition, the fact that indoor pollution sources and ventilation have a large effect on indoor PM_2.5_ concentration can be seen as a major limitation of the model proposed in this study because these variables are not reflected.

However, in order to propose a practical prediction method and overcome the limitations of limited input variables, it is judged that the indoor and outdoor temperature difference, relative humidity, and the difference between temperature and humidity can be substituted for the ventilation rate and indoor pollutants as input variables. Furthermore, by employing an MLR model that allows us to examine the weights of each variable, we were able to assess the importance of input variables on an hourly basis. When evaluating the performance of the models by time period, the prediction performance of the model during the early morning hours, which corresponded to the sleep duration of occupants (H~H), showed the best results. By contrast, the models trained on time periods when occupants are most active, such as H8 and H19, exhibited poorer prediction performance. These results suggest that indoor pollution sources, not explainable by outdoor variables, might have influenced indoor PM_2.5_ concentrations during those specific time periods. It was evident that time is an essential variable that must be considered when predicting indoor PM_2.5_ concentrations.

Furthermore, to gain a more detailed understanding of the factors influencing the improvement of model performance, we evaluated the model performance by season. The results showed that the seasonal characteristics had a significant impact on indoor PM_2.5_ concentrations and the performance of the prediction models. This study is expected to serve as valuable reference material for future research on predicting indoor PM_2.5_ concentrations.

## Figures and Tables

**Table 1:** Specifications of the measuring device.

Specification	Dust Mon (Sentry Co. Ltd., Seoul, Republic of Korea)	
Appearance	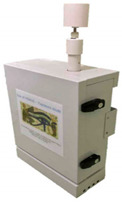	
	300 (W) × 150 (D) × 430 (H) MM, 9 kg	
Metrics	Particulate matter	PM_2.5_
	Other	Temperature, Relative humidity
Measurement Range	Particulate matter	0–100,000 µg/m^3^
Flux	0.5 L/min	
Operating range	−30 °C~60 °C, 0~99% relative humidity (RH)	
Working power	220 VAC/60 Hz	
Power	144 kW/month	
Communications	LTE Cat M1	
Data storage	SD CARD	

**Table 2:** Distribution of indoor and outdoor measurement variables.

Variable	Units	I/O	N	Mean ± S.D.	Median	Max	I/O Ratio		Skewness	Kurtosis
							Mean	Median		
PM_2.5_	µg/m^3^	Indoor	80,572	10.31 ± 13.70	6.00	460.56	0.39	0.29	4.44	50.27
		Outdoor	80,572	26.28 ± 20.69	21.00	227.00			1.76	5.38
Temperature	°C	Indoor	80,572	27.01 ± 2.61	27.00	33.30	2.15	2.16	0.28	−0.36
		Outdoor	80,572	12.55 ± 10.65	12.50	40.00			−0.06	−0.80
Relative humidity	%	Indoor	80,572	46.29 ± 18.61	41.06	94.25	0.61	0.48	1.39	1.18
		Outdoor	80,572	75.37 ± 22.89	86.19	99.90			−0.91	−0.42

**Table 3:** The results of the MLR model.

Model	N (Train/Test)	Coefficients						Intercept.	RMSE	MAE	R^2^
		a	b	c	d	e	f				
Previous method	72,300 (50,610/ 21,690)	16.44	−9.44	4.46	−0.71	−4.57	0.69	9.62	4.86594	3.66157	0.25

**Table 4:** The results of the MLR model separated per hour.

Model	N (Train/Test)	RMSE	MAE	R^2^	Model	N (Train/Test)	RMSE	MAE	R^2^
H0	2113/906	4.41158	3.36610	0.25	H12	2097/899	5.12931	3.72021	0.28
H1	2153/923	4.57719	3.54399	0.31	H13	2116/907	5.16333	3.78103	0.24
H2	2204/945	4.49406	3.41214	0.31	H14	2109/905	4.23131	3.13249	0.24
H3	2223/953	4.65165	3.53079	0.29	H15	2073/889	3.34157	2.55109	0.33
H4	2199/942	4.75141	3.60395	0.34	H16	2072/889	3.94571	2.91574	0.28
H5	2199/943	4.61411	3.57164	0.30	H17	2064/885	4.39213	3.26841	0.25
H6	2137/916	4.80890	3.69874	0.31	H18	2093/897	4.70427	3.61987	0.28
H7	2132/914	6.50780	5.01471	0.25	H19	2143/919	5.65613	4.12237	0.20
H8	2140/918	7.17099	5.37863	0.22	H20	2133/915	5.59225	4.18854	0.24
H9	2136/916	6.87636	5.08694	0.33	H21	2089/896	5.10041	3.79425	0.25
H10	2150/922	6.57170	4.95590	0.31	H22	2074/889	4.89382	3.64940	0.28
H11	2141/918	5.64699	4.10089	0.25	H23	2077/891	4.54320	3.38853	0.24

**Table 5:** The results of the MLR model separated per hour.

Model	Coefficients						Intercept.	Model	Coefficients						Intercept.
	a	b	c	d	e	f			a	b	c	d	e	f	
H0	14.00	−6.45	5.83	−0.29	−0.16	−0.19	4.47	H12	8.22	−5.21	5.64	−1.97	−3.61	0.22	6.17
H1	13.62	−6.41	6.76	−0.56	0.07	−0.73	3.50	H13	9.78	−3.63	4.67	−2.27	−4.25	−0.84	6.56
H2	9.86	−6.92	5.83	0.27	−0.38	0.03	4.05	H14	6.76	−3.86	4.69	−1.45	−4.53	−0.33	6.20
H3	8.92	−8.17	5.63	1.40	−0.86	0.84	4.04	H15	4.98	−3.22	3.35	−1.72	−5.28	−0.87	6.75
H4	10.06	−8.50	5.27	0.19	−0.03	0.17	4.70	H16	5.72	−2.66	3.61	−1.53	−4.41	−0.56	5.77
H5	9.60	−9.36	4.81	3.31	−0.40	0.95	5.15	H17	6.86	−2.87	3.17	−0.82	−6.02	−1.26	6.91
H6	8.70	−8.01	5.12	2.45	0.65	0.3	3.84	H18	9.11	−3.61	3.09	−2.23	−6.92	−0.58	8.55
H7	14.73	−7.58	5.27	0.34	2.45	−0.86	5.19	H19	10.91	−4.63	4.37	−2.61	−3.76	−0.06	6.74
H8	15.70	−15.77	9.09	−0.56	−2.23	3.74	9.27	H20	9.51	−4.59	5.90	−2.81	−3.04	0.40	5.86
H9	12.15	−17.11	9.56	0.59	−3.58	5.12	9.85	H21	10.07	−5.07	4.99	−3.10	−1.95	0.16	6.02
H10	12.12	−13.90	7.43	0.03	−4.47	1.67	11.85	H22	10.26	−4.39	5.82	−2.87	−0.76	−1.06	4.30
H11	8.84	−9.03	5.49	−1.72	−4.51	0.79	10.18	H23	10.68	−4.83	5.35	−1.40	−1.64	−0.43	4.42

**Table 7:** RMSE of the proposed model by season.

Model	Year	Spring	Summer	Fall	Winter
H0	4.41158	12.34248	6.96156	6.56937	15.74986
H1	4.57719	11.13504	7.01424	6.83583	15.80936
H2	4.49406	8.10813	8.29448	7.56207	13.25280
H3	4.65165	11.32884	10.50381	6.68133	11.75929
H4	4.75141	9.55735	13.42097	7.39000	9.87304
H5	4.61411	9.58722	14.30802	6.70083	9.71402
H6	4.80890	14.22191	13.67163	9.14738	12.81491
H7	6.50780	14.79353	18.09313	11.90778	17.34678
H8	7.17099	17.38503	13.09771	8.77013	20.58378
H9	6.87636	18.37358	15.05030	12.30992	21.72775
H10	6.57170	13.15244	13.09226	8.31716	19.67714
H11	5.64699	11.99965	11.74966	7.32458	17.13957
H12	5.12931	12.72795	12.71104	6.55808	15.19633
H13	5.16333	12.26430	14.66854	8.40014	13.69871
H14	4.23131	9.99186	13.51807	8.57123	12.54090
H15	3.34157	14.57501	15.19851	6.61890	16.26682
H16	3.94571	14.08520	14.94630	5.25771	13.67740
H17	4.39213	10.96050	13.81440	20.00288	12.47072
H18	4.70427	11.03774	12.46243	11.41809	14.39600
H19	5.65613	9.94045	8.85166	17.84064	15.55853
H20	5.59225	14.90723	11.33855	13.60358	21.36625
H21	5.10041	11.61009	8.888941	7.98565	16.00126
H22	4.89382	13.85268	9.27154	7.68929	17.13908
H23	4.54320	13.696636	9.81764	7.23575	16.53751

## Data Availability

Not applicable.

## References

[1] (2019). Korean Exposure Factors Handbook.

[2] F KSOSTAT (2022). Kindicator. http://www.index.go.kr/unify/idx-info.do?idxCd=4275#:~:text=%EA%B5%AD%EC%A0%9C%EC%A0%81%EC%9C%BC%EB%A1%9C%20%ED%95%9C%EA%B5%AD%EC%9D%98%20%EB%AF%B8%EC%84%B8,2%EB%B0%B0%20%EC%A0%95%EB%8F%84%20%EC%8B%AC%ED%95%9C%20%EA%B2%83%EC%9D%B4%EB%8B%A4.

[3] Wang X., Xu Z., Su H., Ho H.C., Song Y., Zheng H., Hossain M.Z., Khan M.A., Bogale D., Zhang H. (2021). Ambient Particulate Matter (PM_1_, PM_2.5_, PM_10_) and Childhood Pneumonia: The Smaller Particle, the Greater Short-Term Impact?. Sci. Total Environ..

[4] Schwartz J. (2000). Harvesting and Long Term Exposure Effects in the Relation between Air Pollution and Mortality. Am. J. Epidemiol..

[5] Franklin M., Koutrakis P., Schwartz J. (2008). The Role of Particle Composition on the Association between PM_2.5_ and Mortality. Epidemiology.

[6] Chen L.Y., Tsay Y.S., Jung C.C. Machine Learning Models for Indoor PM_2.5_ Concentrations in Residential Architecture in Taiwan. Proceedings of the CLIMA 2022 Conference.

[7] Jung J., Ahn J. (2019). Intelligent User Pattern Recognition Based on Vision, Audio and Activity for Abnormal Event Detections of Single Households. J. Korea Soc. Comput. Inf..

[8] Lee S.H., Yoon Y.A., Jung J.H., Chang T.W., Kim Y.S. (2020). A Machine Learning Model for Predicting Silica Concentrations through Time Series Analysis of Mining Data. J. Korean Soc. Qual. Manag..

[9] Wei W., Ramalho O., Malingre L., Sivanantham S., Little J.C., Mandin C. (2019). Machine Learning and Statistical Models for Predicting Indoor Air Quality. Indoor Air.

[10] Choi Y.J., Choi E.J., Cho H.U., Moon J.W. (2021). Development of an Indoor Particulate Matter (PM_2.5_) Prediction Model for Improving School Indoor Air Quality Environment. KIEAE J..

[11] Lagesse B., Wang S., Larson T.V., Kim A.A. (2020). Predicting PM_2.5_ in Well-Mixed Indoor Air for a Large Office Building Using Regression and Artificial Neural Network Models. Environ. Sci. Technol..

[12] Choi Y., Choi E., Cho H., Moon J. (2021). Development of a Prediction Model for Indoor Fine Dust (PM_2.5_) to Improve Indoor Air Quality in School Facilities. KIEAE J..

[13] Phillips J.L., Field R., Goldstone M., Reynolds G.L., Lester J.N., Perry R. (1993). Relationships between indoor and outdoor air quality in four naturally ventilated offices in the United Kingdom. Atmos. Environ. Part A Gen. Top.

[14] Ji W., Zhao B. (2015). Contribution of Outdoor-Originating Particles, Indoor-Emitted Particles and Indoor Secondary Organic Aerosol (SOA) to Residential Indoor PM_2.5_ Concentration: A Model-Based Estimation. Build. Environ..

[15] Liu D.L., Nazaroff W.W. (2001). Modeling Pollutant Penetration Across Building Envelopes. Atmos. Environ..

[16] Bakht A., Han S., Khan M.S., Jang K., Kim K.H. (2022). Deep Learning-Based Indoor Air Quality Forecasting Framework for Indoor Subway Station Platforms. Toxics.

[17] Marzouk M., Atef M. (2022). Assessment of Indoor Air Quality in Academic Buildings Using IoT and Deep Learning. Sustainability.

[18] AirKorea. https://www.airkorea.or.kr/index.

[19] Chen C., Zhao B. (2011). Review of Relationship between Indoor and Outdoor Particles: I/O Ratio, Infiltration Factor and Penetration Factor. Atmos. Environ..

[20] Kang J.-W., An C.-J., Choi W. (2020). Surrounding environment and indoor fine dust concentration distribution characteristics based on indoor/outdoor concentration ratio (I/O ratio): Focusing on previous research reviews and measurement results in Busan and Pyeongtaek elementary schools in summer. J. Korean Soc. Remote Sens..

[21] Abdipour M., Ramazani S.H.R., Younessi-Hmazekhanlu M., Niazian M. (2018). Modeling Oil Content of Sesame (*Sesamum indicum* L.) Using Artificial Neural Network and Multiple Linear Regression Approaches. J. Am. Oil Chem. Soc..

[22] Emamgholizadeh S., Parsaeian M., Baradaran M. (2015). Seed Yield Prediction of Sesame Using Artificial Neural Network. Eur. J. Agron..

[23] May R.J., Dandy G.C., Maier H.R. (2011). Review of Input Variable Selection Methods for Artificial Neural Networks. Artificial Neural Networks—Methodological Advances and Biomedical Applications.

[24] Czernecki B., Półrolniczak M., Kolendowicz L., Marosz M., Kendzierski S., Pilguj N. (2017). Influence of the Atmospheric Conditions on PM_10_ Concentrations in Poznań, Poland. J. Atmos. Chem..

[25] Xu C., Xu D., Liu Z., Li Y., Li N., Chartier R., Li N. (2020). Estimating Hourly Average Indoor PM_2.5_ Using the Random Forest Approach in Two Megacities, China. Build. Environ..

[26] Yeom M.-S., Cho G.-Y. (2007). Natural Ventilation of a High-Rise Residential Building Using a Double Skin System. Archit.

[27] Isixsigma Variance Inflation Factor (VIF). https://www.isixsigma.com/dictionary/variance-inflation-factor-vif/.

[28] Jeon Y.T., Yu S.H., Kwon H.Y. (2020). Improvement of PM Forecasting Performance by Outlier Data Removing. J. Korea Multimed. Soc..

[29] Kashi H., Emamgholizadeh S., Ghorbani H. (2014). Estimation of Soil Infiltration and Cation Exchange Capacity Based on Multiple Regression, ANN (RBF, MLP), and ANFIS Models. Commun. Soil Sci. Plant Anal..

[30] Masood A., Ahmad K. (2020). A Model for Particulate Matter (PM_2.5_) Prediction for Delhi Based on Machine Learning Approaches. Procedia Comput. Sci..

[31] Scikit-Learn. Sklearn.linear_model.SGDRegressor. https://scikit-learn.org/stable/modules/generated/sklearn.linear_model.SGDRegressor.html.

[32] Kang M.J. (2020). Comparison of Gradient Descent for Deep Learning. J. Korea Acad. Ind. Coop. Soc..

[33] Konečný J., Liu J., Richtárik P., Takáč M. (2015). Mini-Batch Semi-Stochastic Gradient Descent in the Proximal Setting. IEEE J. Sel. Top. Signal Process..

[34] Taşan S., Demir Y. (2020). Comparative analysis of MLR, ANN, and ANFIS models for prediction of field capacity and permanent wilting point for Bafra plain soils. Commun. Soil Sci. Plant Anal..

[35] Nishihama Y., Ishikuro M., Miyashita C., Yamamoto-Hanada K., Sato M., Kato N., Minami Y., Hori T., Doi H., Araki A. (2021). Indoor Air Quality of 5000 Households and Its Determinants. Part A: Particulate Matter (PM_2.5_ and PM_10–2.5_) Concentrations in the Japan Environment and Children’s Study. Environ. Res..

[36] National Institute of Environmental Research Annual Report of Air Quality in Korea 2021. https://www.niehs.nih.gov/research/programs/geh/partnerships/network/centres/south_korea/index.cf.

[37] Ministry of Environment Implementation Regulations of Indoor Air Quality Management Act. Year. https://www.law.go.kr/%EB%B2%95%EB%A0%B9/%EC%8B%A4%EB%82%B4%EA%B3%B5%EA%B8%B0%EC%A7%88%EA%B4%80%EB%A6%AC%EB%B2%95%EC%8B%9C%ED%96%89%EA%B7%9C%EC%B9%99.

[38] Park B.R., Choi D.H., Kang D.H. (2020). Seasonal Contribution of Indoor Generated and Outdoor Originating PM_2.5_ to Indoor Concentration Depending on Airtightness of Apartment Units. J. Archit. Inst. Korea Struct. Constr..

[39] Park J., Kim E., Choe Y., Ryu H., Kim S., Woo B.L., Cho M., Yang W. (2020). Indoor to Outdoor Ratio of Fine Particulate Matter by Time of the Day in House According to Time-activity Patterns. J. Environ. Health Sci..

[40] Park S., Yoon D., Kong H., Kang S., Lee C. (2021). A Case Study on Distribution Characteristics of Indoor and Outdoor Particulate Matters (PM_10_, PM_2.5_) and Black Carbon (BC) by Season and Time of the Day in Apartment. J. Environ. Health Sci..

[41] Han Y., Qi M., Chen Y., Shen H., Liu J., Huang Y., Chen H., Liu W., Wang X., Liu J. (2015). Influences of Ambient Air PM_2.5_ Concentration and Meteorological Condition on the Indoor PM_2.5_ Concentrations in a Residential Apartment in Beijing Using a New Approach. Environ. Pollut..

[42] Zhao L., Chen C., Wang P., Chen Z., Cao S., Wang Q., Xie G., Wan Y., Wang Y., Lu B. (2015). Influence of Atmospheric Fine Particulate Matter (PM_2.5_) Pollution on Indoor Environment during Winter in Beijing. Build. Environ..

[43] Qi M., Zhu X., Du W., Chen Y., Chen Y., Huang T., Pan X., Zhong Q., Sun X., Zeng E.Y. (2017). Exposure and Health Impact Evaluation Based on Simultaneous Measurement of Indoor and Ambient PM_2.5_ in Haidian, Beijing. Environ. Pollut..

[44] Kearney J., Wallace L., MacNeill M., Heroux M.-E., Kindzierski W., Wheeler A. (2014). Residential Infiltration of Fine and Ultrafine Particles in Edmonton. Atmos. Environ..

[45] Yang S., Mahecha S.D., Moreno S.A., Licina D. (2022). Integration of Indoor Air Quality Prediction into Healthy Building Design. Sustainability.

[46] Yuchi W., Gao J., Xie W., Wang L., Zhang Z., Lai A.C.K. (2019). Evaluation of Random Forest Regression and Multiple Linear Regression for Predicting Indoor Fine Particulate Matter Concentrations in a Highly Polluted City. Environ. Pollut..

[47] Elbayoumi M., Ramli N.A., Yusof N.F.F.M., Yahaya A.S.B., Al Madhoun W., Ul-Saufie A.Z. (2014). Multivariate Methods for Indoor PM_10_ and PM_2.5_ Modelling in Naturally Ventilated Schools Buildings. Atmosphere.

[48] Zhang H., Srinivasan R., Yang X. (2021). Simulation and Analysis of Indoor Air Quality in Florida Using Time Series Regression (TSR) and Artificial Neural Networks (ANN) Models. Symmetry.

[49] Zhang H., Wang Y., Yang X., Wang J., Xie M., Wei X., Liu H., Yuan Y. (2022). Factors Influencing Indoor Air Pollution in Buildings Using PCA-LMBP Neural Network: A Case Study of a University Campus. Build. Environ..

[50] Zhong J., Ding J., Su Y., Shen G., Yang Y., Wang C., Tao S. (2012). Carbonaceous Particulate Matter Air Pollution and Human Exposure from Indoor Biomass Burning Practices. Environ. Eng. Sci..

[51] Abt E., Suh H.H., Catalano P., Koutrakis P. (2000). Relative Contribution of Outdoor and Indoor Particle Sources to Indoor Concentrations. Environ. Sci. Technol..

[52] Pagel É., Costa Reis N., de Alvarez C.E., Santos J.M., Conti M.M., Boldrini R.S., Kerr A.S. (2016). Characterization of the Indoor Particles and Their Sources in an Antarctic Research Station. Environ. Monit. Assess..

[53] Shrubsole C., Ridley I., Biddulph P., Milner J., Vardoulakis S., Ucci M., Oreszczyn T., Wilkinson P., Marmot A., Davies M. (2012). Indoor PM_2.5_ Exposure in London’s Domestic Stock: Modelling Current and Future Exposures Following Energy Efficient Refurbishment. Atmosphere.

